# Clinical changes of serum melatonin and ICAM-1 levels in patients with basal ganglia hypertensive intracerebral hemorrhage

**DOI:** 10.12669/pjms.39.4.7252

**Published:** 2023

**Authors:** Hongbo Hou, Xin Li

**Affiliations:** 1 Hongbo Hou, Department of Neurosurgery, Suzhou Hospital of Traditional, Chinese and Western Medicine, Suzhou, 215101, Jiangsu Province, P.R. China; 2 Xin Li, Department of Neurosurgery, Suzhou Hospital of Traditional, Chinese and Western Medicine, Suzhou, 215101, Jiangsu Province, P.R. China

**Keywords:** Serum melatonin, Intercellular adhesion molecule-1, Basal ganglia cerebral hemorrhage

## Abstract

**Objective:**

To investigate the clinical changes of serum melatonin and intercellular adhesion molecule-1 (ICAM-1) levels in patients with basal ganglia hypertensive intracerebral hemorrhage (HICH).

**Methods:**

In this retrospective observational study, patients with HICH in Suzhou Hospital of Traditional Chinese and Western Medicine from January 2020 to January 2021 were included as the observation group (n=120), and 120 healthy volunteers who underwent physical examination were included as the control group. According to the Glasgow Coma Scale (GCS), the observation group was divided into four subgroups: normal group (n=27), mild group (n=51), moderate group (n=24), and severe group (n=18). Differences in serum melatonin and ICAM-1 were compared between the observation group and the control group, and between the subgroups. Logistic regression was used to analyze the risk factors of poor prognosis, and the ROC curve was used to analyze the influence of melatonin and ICAM-1 levels on patient prognosis.

**Results:**

Serum melatonin levels were lower in the observation group compared to the control group, while ICAM-1 levels were higher (*P*<0.05). With the aggravation of brain injury, serum melatonin decreased and ICAM increased (*P*<0.05). Decreased serum melatonin and increased ICAM-1 were independent risk factors for poor prognosis in patients with HICH. The combined AUC of serum melatonin and ICAM-1 for the detection of poor prognosis in patients with HICH was 0.860, which was higher than that of the two alone (*P*<0.05).

**Conclusions:**

Low serum melatonin levels and high ICAM-1 levels are associated with poor prognosis in patients with HICH and can be used as predictors of patients’ prognosis.

## INTRODUCTION

Intracerebral hemorrhage (ICH) is the most devastating subtype of stroke with a very high morbidity and mortality rate, usually affecting the basal ganglia, pons, cerebellum, or thalamus.^1^ Basal ganglia hypertensive intracerebral hemorrhage (HICH) refers to the symptoms of hemorrhage in the basal ganglia of the brain.^2^ HICH is mainly caused by hypertension combined with cerebral arteriosclerosis. Specifically, long-term hypertension can cause small blood vessels in the brain to undergo hyaline degeneration and form microaneurysms, which rupture when blood pressure rises, leading to bleeding. 3 If the amount of bleeding is light and the patient is clearheaded, treatment for this mild condition includes dehydration, cranial pressure reduction, rehabilitation function training, etc.^4^ If the amount of bleeding is large, the degree of limb paralysis is severe, and the patient is delirious or even the vital signs are unstable, surgery is required.^4^,^5^ At present, the judgment of the degree of brain damage and prognosis of patients with HICH is mainly based on the amount of hemorrhage, and little is known about the clinical application of laboratory indicators in this disease.^3^,^4^

Melatonin is mainly synthesized and secreted by the pineal gland, which can aggregate melanin particles.^6^ Serum melatonin is the strongest endogenous free radical scavenger and its basic function is to participate in the antioxidant system and prevent cells from oxidative damage.^7^ Animal experiment has shown that melatonin has a good protective effect on ischemic brain injury.^8^ Intercellular adhesion molecule-1(ICAM-1), a transmembrane glycoprotein, participates in physiological and pathological processes such as cell signal transduction and activation, cell tissue growth and differentiation, immune response, inflammatory response, angiogenesis, and tumor metastasis.^9^ Studies have shown that ICAM-1 is closely related to the degree of brain edema in patients with acute intracerebral hemorrhage and to the neurological deficit in patients with intracerebral hemorrhage.^10^,^11^ Both serum melatonin and ICAM-1 have good application potential in patients with cerebral hemorrhage.^9^-^11^ However, there are few studies have investigated the association between melatonin or ICAM-1 and HICH. Therefore, in this study, we aimed to explore the clinical changes in serum melatonin and ICAM-1 levels in patients with HICH.

**Fig.1:**
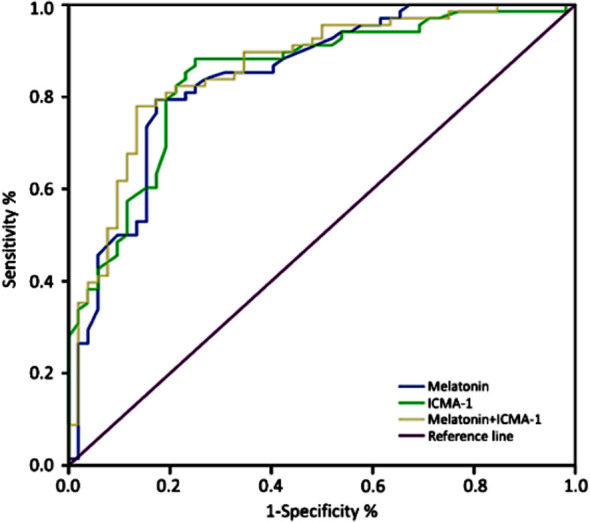
ROC curve.

## METHODS

In this retrospective observational study, patients with HICH treated in Suzhou Hospital of Traditional Chinese and Western Medicine from January 2020 to January 2021 were included as the observation group (n=120). These patients were sub-divided according to the Glasgow Coma Scale (GCS):^12^ normal (n=27), mild (n=51), moderate (n=24), and severe (n=18) groups. Another 120 healthy volunteers who underwent physical examination during the same period were selected as the control group.

### Inclusion criteria:

- All patients in the observation group met the diagnostic criteria for basal ganglia cerebral hemorrhage according to the Chinese guidelines for the diagnosis and treatment of cerebral hemorrhage.^13^
- The time from the first onset to admission was within six hours.
- The basic clinical data were complete, including the test results of serum melatonin and ICAM-1.
- Patients were older than 18.

### Exclusion criteria:

- Patients with cerebrovascular malformations, aneurysms, and other diseases that may cause cerebral hemorrhage.
- Patients who had recently taken anticoagulant drugs and drugs that affect the detection results of serum melatonin and ICAM-1.
- Abnormal liver, kidney, lung, or other organ function.
- Patients with a malignant tumor.

### Ethical Approval:

This study was approved by the medical ethics committee of Suzhou Hospital of Traditional Chinese and Western Medicine (Approval No.: 2022-026; Date: 2022-07-29).

### Data Collection:

Baseline data for all participants were collected including age, gender, smoking history, history of diabetes, history of hypertension, and body mass index (BMI). Serum levels of melatonin and ICAM-1 were analyzed. Specifically, 5ml of venous blood was collected under fasting conditions and placed into a vacuum tube, then the sample was placed into a water tank at 37°C. Once the blood was coagulated, the tube was placed into a centrifuge and rotated at a speed of 3000r/min for 5 mins to separate the serum. Serum melatonin and ICAM-1 were detected in strict accordance with the instructions of the ELISA kit, which was purchased from Shanghai keaibo biological Co., Ltd.

The degree of brain injury was assessed according to the Glasgow Coma Scale (GCS).^12^ The assessment indicators include three items: limb movement (six points), speech response (five points), and eye-opening response (four points). The minimum total score is three points and the maximum is 15 points. The lower the score, the more serious the brain injury, with 15 points representing no impairment of consciousness. A score of 13-14 points indicates mild disturbance of consciousness; 9~12 points, moderate disturbance of consciousness; 3~8 points, serious disturbance of consciousness.

Prognosis according to the Modified Rankin Scale (MRS),^14^ the prognosis of the patients was evaluated after six months of treatment, and a score of 1-2 was regarded as a good prognosis, and a score of three to six was regarded as a poor prognosis.

### Statistical Analysis:

SPSS 22.0 software was used for statistical analysis. Measurement data that were normally distributed with equal variance were represented as (*χ̅*±*S*). T-test was used for comparison between the two groups, and ANOVA was used among multiple groups. Measurement data of skewed distribution or uneven variance were expressed as M (Q_L_, Q_U_), and Z-test was used between the two groups. Counting data were expressed as n (%) and analyzed by *χ^2^* test. Multivariate logistic regression was used to analyze the influencing factors of poor prognosis in patients with HICH. The receiver operating characteristic (ROC) curve was drawn to analyze the value of serum melatonin and ICAM-1 in evaluating the prognosis of patients with HICH. If the *p-Value* was less than 0.05, the result was statistically significant.

## RESULTS

There was no significant difference in the baseline data between the observation group and the control group (*P*>0.05) (Table-I). Serum melatonin was significantly lower in the observation group compared to the control group, while ICAM-1 levels were significantly higher compared to the control group (*P*<0.05) (Table-II). The more serious the disturbance of consciousness, the lower the serum melatonin level and the higher the ICAM-1 level (*P*<0.001) (Table-III).

**Table-I:** Comparison of baseline data between the observation group and control group [n (%)].*χ̅*±*S*].

Item	Observation Group (n=120)	Control Group (n=120)	t/χ^2^	P-Value
Gender (Male, %)	89 (74.17%)	95 (79.17%)	0.839	0.36
Age (years)	56.90±9.20	58.30±10.64	0.368	0.372
BMI (kg/m^2^)	22.96±1.72	22.79±1.65	0.308	0.426
Smoking (yes, %)	47 (39.17%)	51 (42.5%)	0.276	0.599
Diabetes (yes, %)	14 (11.67%)	17 (14.17%)	0.333	0.564
Hypertension (yes, %)	26 (21.67%)	21 (17.5%)	0.663	0.416

**Table-II:** Serum melatonin and ICAM-1 levels between the observation group and control group.

Index	Observation group (n=120)	Control group (n=120)	t	p-Value
Melatonin (ng/ml)	36.15±6.02	70.94±4.45	-50.915	<0.001
ICAM-1(ng/mL)	277.75±46.00	213.02±34.55	12.325	<0.001

**Table-III:** Serum melatonin and ICAM-1 levels in patients with different degrees of consciousness disorders.

Degree of disturbance of consciousness	Serum melatonin (ng/mL)	ICAM-1 (ng/mL)
Normal-group (n=27)	42.31±4.22	237.15±28.95
Mild-group (n=51)	37.67±3.35^a^	265.06±39.80^a^
Moderate-group (n=24)	32.91±2.76^a,b^	306.08±17.16^a,b^
Severe-group (n=18)	26.96±3.38^a,b,c^	336.89±23.10^a,b,c^
*F*	80.694	44.65
*p*-Value	<0.001	<0.001

***Note:*** a indicates that compared with the normal group, *p*<0.05; b indicates that compared with the mild group, *p*<0.05; c indicates that compared with the moderate group, *p*<0.05.

After six months of treatment, 68 patients had good prognosis, and 52 patients had poor prognosis. Multivariate logistic regression analysis showed that lower serum melatonin and higher ICAM-1 levels were risk factors for poor prognosis in patients with HICH (*P*<0.05). The AUC of serum melatonin and ICAM-1 in independently predicting the adverse prognosis of patients with HICH were 0.845 and 0.849, respectively. The AUC predicted by the combined detection of the two indicators was 0.863, which was higher than single detection alone (*P*<0.05) (Table-V). The sensitivity and specificity were 89.7% and 55.1% respectively.

**Table-IV:** Risk factors for poor prognosis in patients with hICH.

Index	B	S.E.	Wald χ^2^	p-Value	OR	95% CI
Serum melatonin	-0.145	0.07	4.351	0.037	1.156	1.009~1.326
ICAM-1	0.022	0.009	6.074	0.014	0.978	0.961~0.995

***Note:*** B indicates partial regression system; S.E. indicates standard error; Wald χ^2^ (B/S.E.)2; OR is odds ratio; 95%CI is the confidence interval of OR.

**Table-V:** Prognostic value of serum melatonin and ICAM-1 in patients with hICH.

Index	AUC	95%CI	Cut-off Value	Sensitivity (%)	Specificity (%)	p-Value
Serum melatonin	0.844	0.771~0.916	36.55	79.4	64.1	0.037
ICAM-1	0.846	0.775~0.917	286	88.2	63.2	0.036
Joint detection	0.860	0.792~0.928	11.14	89.7	55.1	0.035

## DISCUSSION

The results from this study showed that lower serum melatonin levels and higher ICAM-1 levels are risk factors for poor prognosis in patients with HICH. Previous research revealed that melatonin can reduce secondary brain damage after cerebral hemorrhage and protect brain tissue by affecting cell apoptosis, inflammation, oxidative stress, DNA damage, brain edema, blood-brain barrier damage, and reducing the opening of mitochondrial membrane permeability transition pore.^15^

In our study, we found that the level of serum melatonin in patients with HICH was low, and the more serious the condition was, the lower the expression level was, indicating that serum melatonin was closely related to the degree of brain injury in HICH patients. A large amount of experimental evidence shows that melatonin may be a neuroprotective agent for acute brain injury and chronic neurodegenerative diseases.^16^-^18^ Abu-Elfotuh K^19^ repeatedly tested the neuroprotective effect of melatonin in different experimental models of intracerebral hemorrhage. Liang F et al.^20^ pointed out that melatonin can alleviate mitochondrial damage of neurons by activating the PPARδ/PGC-1α pathway, so as to reduce brain damage.

In addition, the results of this study showed that serum melatonin is a risk factor affecting the poor prognosis of patients with HICH, the lower the content of melatonin, the worse the prognosis of patients, which is contrary to the findings of Lorente et al. as they found higher serum melatonin levels in non-surviving patients than in surviving patients with spontaneous intracerebral hemorrhage (SIH).^21^ We supposed it may be related to the different physical conditions of the participants in these studies as in Lorente’s study were patients with supratentorial intracerebral hemorrhage and clinically severe admitted to intensive care units. Moreover, although the most common cause of basal ganglia hemorrhage is hypertension, not all SIH caused by hypertension. Furthermore, studies focused on HICH should be conducted.

ICAM-1 is mainly expressed in vascular endothelial cells, but its high expression is induced by the response to inflammatory stimulation in epithelial cells and immune cells, which affects the integrity of blood-cerebrospinal fluid barrier.^22^ The results of this study showed that the level of ICAM-1 was higher in patients with HICH, and the higher the degree of brain injury, the higher the level of ICAM-1 expression, suggesting that ICAM-1 is associated with HICH, which basically in line with the post-hoc analysis by Witsch et al.^23^ Meanwhile, ICAM-1 is a risk factor affecting the poor prognosis of patients with HICH. Bhowmick S et al^24^ have shown that the neural complications caused by traumatic brain injury can be treated by blocking the activation of ICAM-1, so as to reduce the migration of immune cells to the brain, neuroinflammation, and cell death, confirming that ICAM-1 levels correlate with the severity of patients with brain injury. The animal experiments by Abadier M *et al*^25^ showed that the ICAM-1 level of endothelial cells is the most critical factor to guide cd4+ TEM cells to move through the blood-brain barrier. Additionally, high concentrations of phosphatidyl ethanol, D-dimer, and other laboratory indicators are high-risk factors for patients with cerebral hemorrhage.^26^,^27^ This study ignores these factors, which may lead our model to underestimate the risk of HICH. This study also showed that serum melatonin and ICAM-1 could be used as indicators to predict the adverse prognosis of patients with HICH, and the predictive effect of their combined detection was better than when used independently.

### Limitations:

First, the current physical and mental state of the patients could affect the authenticity and accuracy of the past data report. Secondly, this study did not focus on all possible detection indicators, limiting the potential clinical scope of the related risk factors of patients with HICH. Last, the sample size of this study was small, and there may be sampling bias. Future studies should expand the scope of the study population, such as increasing the number of subjects and their therapeutic areas, to enhance the clinical implications.

## CONCLUSION

In conclusion, low serum melatonin level and high ICAM-1 level in patients with HICH were found to be related to the degree of brain injury and are influencing factors of poor prognosis in HICH patients. These indicators can be used as predictors of clinical prognosis for patients with HICH, with enhanced detection using both indicators simultaneously.

### Authors’ contributions:

**HH**: conceived and designed the study.

**HH and XL:** collected the data and performed the analysis.

**HH**: was involved in the writing of the manuscript and is responsible for the integrity of the study.

All authors have read and approved the final manuscript.
